# Steel-Reinforced Polyurethane with Mineral Interlayer for Masonry Protection: Laboratory Tests

**DOI:** 10.3390/ma18030503

**Published:** 2025-01-22

**Authors:** Łukasz Hojdys, Piotr Krajewski, Arkadiusz Kwiecień

**Affiliations:** 1 Faculty of Civil Engineering, Cracow University of Technology, Warszawska 24, 31-155 Krakow, Poland; 2 FlexAndRobust Systems Ltd., Warszawska 24, 31-155 Krakow, Poland; ak@flexandrobust.com

**Keywords:** strengthening system, FRPU, fiber-reinforced polyurethane, SRPU, single-lap shear test masonry, bond strength

## Abstract

This paper presents the results of an experimental investigation on a steel-reinforced polyurethane (SRPU) composite system with a mineral interlayer, designed for the protection of existing structures. The composite SRPU was reinforced with unidirectional steel textile embedded in polyurethane matrix PS. In the study, SRPU was applied to a brick substrate via a layer of lime- or cement-based mortar of a thickness of 3 mm, 6 mm, or 10 mm. Single-lap shear tests (SLSTs) were carried out on specimens with and without a mortar interlayer. The reference specimens without a mineral interlayer carried higher loads than the specimens with an interlayer. An increase in the interlayer thickness reduced the shear bond strength. The stiffness of the bond under shear of the tested systems was unaffected by the presence of the mineral interlayer. The mechanical properties of the applied mortars influenced the observed failure modes. The tested SRPU system demonstrated notable efficiency in monotonic testing, outperforming previously reported results.

## 1. Introduction

Strengthening building structural envelopes is one of the key repair issues for damaged buildings. It is the first step in the repair process when considering thermal improvements for nearly zero-energy buildings. Nowadays, heritage buildings also undergo transformations to enhance their thermal comfort. Thus, the use of composite strengthening in the repair and thermal adaptation of historical structures to meet new standards has become a subject of scientific research. The development of composite strengthening systems for heritage structures has been significantly influenced by initiatives from RILEM, particularly those focusing on masonry structures. Since the early 2000s, a diverse group of researchers, conservators, authorities, manufacturers, and other stakeholders has engaged in both experimental and analytical studies to evaluate the effectiveness of various composite materials for reinforcing masonry structures. This extensive body of work has contributed to a growing understanding of composite strengthening techniques, leading to their increasing application in the conservation of real heritage structures, particularly in Italy. The adoption of composite systems has proven beneficial for enhancing the structural integrity of masonry buildings, offering protection against further deterioration induced by static and dynamic loads. The evolution of composite strengthening began with the use of fiber-reinforced polymers (FRPs) and steel-reinforced polymers (SRPs), both of which are composed of high-strength fibers embedded in rigid, high-strength epoxy matrices. These materials were initially evaluated by RILEM TC 223 MSC [1]. However, limitations related to the durability of FRP systems and their incompatibility with masonry substrates [2,3] prompted further investigations into alternatives that utilized mineral matrices, such as cement-based or lime-based mortars [4]. Research by RILEM TC 250 CSM demonstrated the practical viability of these mineral-based composites [5,6,7,8,9,10], and their development has since facilitated their market introduction as strengthening solutions for heritage masonry. The findings from this research have affirmed the effectiveness of these composite materials and their compatibility with weaker masonry substrates [11,12,13,14]. These systems, commonly known as fiber-reinforced cementitious mortars (FRCMs) or textile-reinforced mortars (TRMs), have seen widespread use. However, concerns regarding their long-term durability have prompted further studies to assess the impact of various aging factors on their performance [15]. Current research under RILEM TC 290 IMC continues to address these issues [16,17]. Ghiassi [18] has outlined testing methodologies for FRCM and TRM systems, along with ongoing investigations in this field. A significant challenge for these composites is their resistance to repeated dynamic and cyclic loading, which is particularly critical in seismic regions [19]. In such areas, the robustness of these materials is often enhanced through the use of special connectors [20,21]. However, the irreversible damage caused by drilling holes for these connectors is typically not accepted by heritage authorities, especially when it affects the façades of historical buildings.

An alternative approach that addresses some of the drawbacks associated with FRCM and TRM systems is the use of fiber-reinforced polyurethane (FRPU) systems. These systems utilize high-strength fibers embedded in a flexible polyurethane matrix. The seismic performance of FRPU systems has been demonstrated through various tests, including push-over shear tests [22], full-scale shake table tests [23], and forced harmonic vibration tests [24]. The flexibility of the polyurethane matrix allows FRPU systems to absorb and dissipate energy, enabling them to withstand large deformations and high cyclic loads, which is particularly advantageous in seismic regions [25]. Furthermore, the flexibility of the matrix reduces stress concentrations and distributes them over a larger area, thereby enhancing the protection of weak masonry [26]. Notably, even if the composite system detaches from the masonry, it can be reinstalled with a new flexible polyurethane layer, maintaining the effectiveness of the system [27].

The advantages of FRPU systems extend beyond seismic protection, as they also offer benefits in terms of thermal stability [28] and reversibility [29,30]. The long-term stability of the polyurethane matrix and the bond strength of the FRPU system have been studied and shown to remain reliable under aging conditions [31]. These properties make FRPU systems an attractive option for the protection of heritage masonry structures. In particular, combining FRPU with a mineral interlayer ensures compatibility with the masonry and enhances the reversibility of the system, making it a promising solution for seismic regions. This solution allows for the rapid application of the FRPU system without the need to remove plaster from the masonry walls. Unlike FRCM, FRP, or TRM systems, which may fail due to energy accumulation in micro-cracks under repetitive seismic shocks, the FRPU system with a mineral interlayer offers faster curing, ductile behavior, and superior composite performance.

**Figure 1:**
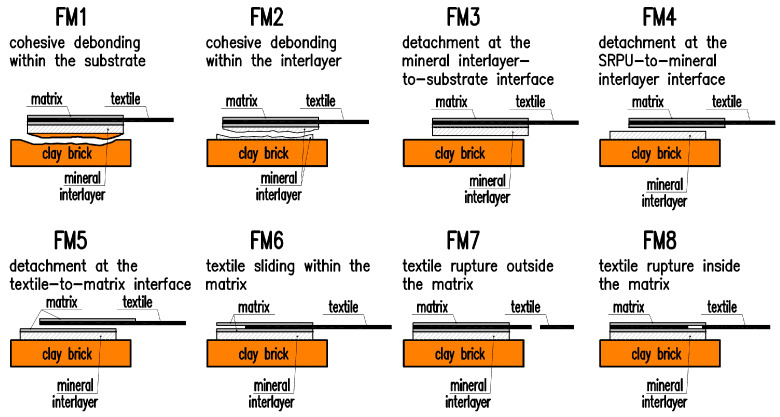
Extended classification of failure modes for specimens with mineral interlayer given in [33].

An important issue regarding the effectiveness of the applied composite strengthening system is ensuring a proper bond between the composite and the substrate. When composite materials are considered as strengthening masonry structures, in addition to determining the tensile strength of the composite, the relationship between axial stress and reinforcement slip should be determined. This relationship describes the bond behavior of the tested system, i.e., the way tensile stress is transferred from the composite reinforcement to the structure. A standardized shear bond test method (known as the single-lap shear test (SLST)) to characterize the composite-to-substrate bond behavior was presented in [6]. This method is commonly used in studies of FRP, FRCM, and FRPU composites, where, in addition to determining the stress–strain curve, the observed failure mode must also be recorded in the research report. The previous studies have shown that the observed failure modes depend on the type of system used, particularly on the mechanical properties of the substrate, the composite matrix, the type and the ratio of reinforcement used, and the bond length [32]. For FRCM materials, six main failure modes have been identified: cohesive debonding in the substrate, detachment at the matrix-to-substrate interface, detachment at the textile-to-matrix interface, sliding of the textile within the matrix, tensile failure of the textile out of the matrix, and tensile failure of the textile within the matrix [6]. In the case of FRP composites on brick substrates, failure most often occurred due to fiber rupture or cohesive debonding in the substrate [1]. The shear bond strength of FRPU composites was studied, among others, in [26,27,31]. These studies identified similar failure modes to those observed for FRCM materials. The use of a mineral layer between the FRPU composite and the substrate may influence the possible failure modes, and some additional failure modes could be observed. In [33], an extension of the failure mode classification used for FRCM and FRPU composites was proposed. A total of eight possible failure modes in FRPU systems with a mineral layer between the composite and the masonry were suggested. A schematic of the failure modes along with descriptions is presented in Figure 1.

This paper presents the results of shear bond tests conducted on the steel-reinforced polyurethane (SRPU) system combined with a mineral interlayer (SRPUI). The pilot study presented in the conference materials [33] analyzed one type of specimens. In comparison to [33], this article considers six different FRPU configurations with a mineral interlayer and their impact on bond behavior. Three mortar layer thicknesses of 3 mm, 6 mm, and 10 mm were used. Furthermore, two types of mortar were analyzed for each thickness: a weaker NHL lime mortar and a stronger cement mortar. The objective of the tests was to assess the influence of both the interlayer thickness and the type of mortar on the failure stress and to identify the possible failure modes.

## 2. Materials and Methods

In the presented study, the shear bond strength of the FRPU system to clay brick was investigated. A mineral mortar layer was applied between the FRPU composite layer and the brick. The geometry of the tested specimens and the testing setup were in accordance with the requirements specified in [6].

### 2.1. Materials

**Figure 2:**
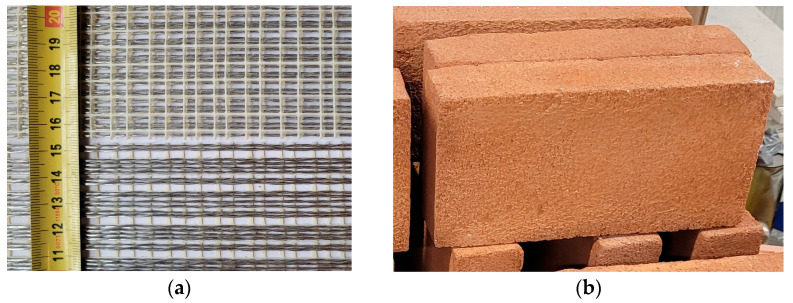
Materials used in the test: (**a**) unidirectional steel fiber textile; (**b**) clay brick.

The FRPU system used consisted of steel textile reinforcement and a polyurethane matrix. The steel textile was made with unidirectionally arranged ultra-high tensile strength steel (UHTSS) cords, which were under investigation in [3,11,12,14] (Figure 2a). The steel textile used has the following characteristics, as provided by the manufacturer: surface mass density of 2000 g/m^2^ and design thickness of 0.254 mm. Each cord is composed of five galvanized (zinc-coated) wires, each with a cross-sectional area of 0.1076 mm^2^. The main mechanical properties of the textile were reported in [5] as follows: tensile strength of 3201 N/mm^2^, ultimate strain of 2.24%, and tensile modulus of elasticity of 186 kN/mm^2^. For the preparation of the specimens, strips of steel textile with an effective width of 50.8 mm were used (24 strands). The composite reinforcement had a cross-sectional area of 12.9 mm^2^/50.8 mm.

The matrix of the composite was made of highly deformable PS-type polyurethane with the following properties: tensile strength of 2.87 N/mm^2^, ultimate strain of 45%, and modulus of elasticity of 14.8 N/mm^2^. It also exhibits hyperelastic behavior in tensile stress–strain relationships [27] and has a water vapor resistance factor ranging between 1200 and 1300. The resulting composite, which combines steel cords (UHTSS) with flexible polyurethane, is referred to as steel-reinforced polyurethane (SRPU) [23,26].

In this study, solid clay bricks from SanMarco-Terreal (Noale, Italy) (Figure 2b) were used. These bricks have dimensions of 120 mm × 250 mm × 55 mm, a compressive strength of 14.8 N/mm^2^, a modulus of elasticity of 5.76 kN/mm^2^, and a tensile strength of 2.5 N/mm^2^ [6].

The SRPU strip was applied directly onto the brick (specimens labeled as R) or via a layer of mortar. For the specimens labeled as L, a lime-based mortar was used, while for the specimens labeled as C, a cement-based mortar was applied. Three thicknesses of the mineral interlayer were considered: 3 mm, 6 mm, and 10 mm (Table 1). The assumed mortar thicknesses were similar to those of the plaster or mineral layer used in fiber-reinforced cementitious matrix (FRCM) and textile-reinforced mortar (TRM) systems. After 28 days of curing, the compressive strengths of the mortars were 7.2 N/mm^2^ for the lime-based mortar and 32.6 N/mm^2^ for the cement-based mortar [34]. The elastic moduli of the mortars used, based on the data provided by the manufacturer, were 16 GPa and 10 GPa for the lime mortar and cement mortar, respectively. The water vapor resistance factors for these mortars ranged from 5 to 20.

### 2.2. Specimen Preparation

**Figure 3:**
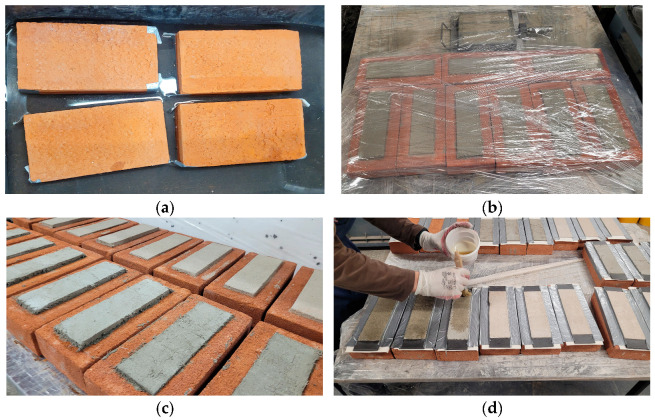

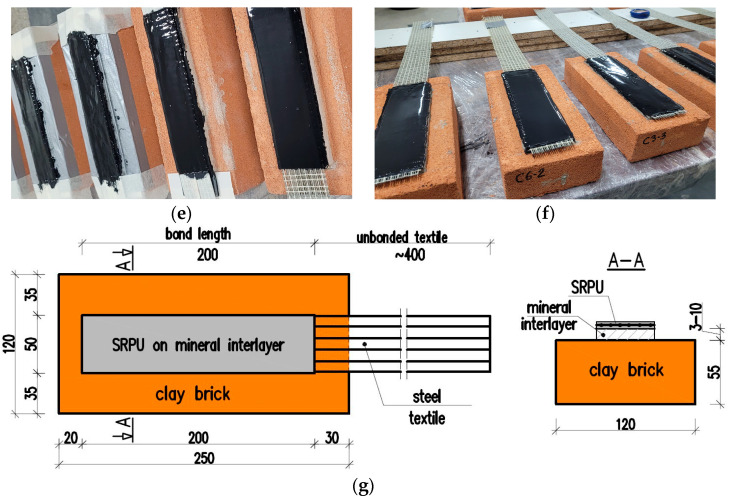
Preparation of the specimens: (**a**) wetting clay bricks; (**b**) curing of the specimens after mineral interlayer application; (**c**) bricks with mineral interlayer; (**d**) application of primer; (**e**) specimens after application of SRPU layer; (**f**) specimens after removal of protective tapes; (**g**) specimen geometry, dimensions in mm.

**Figure 4:**
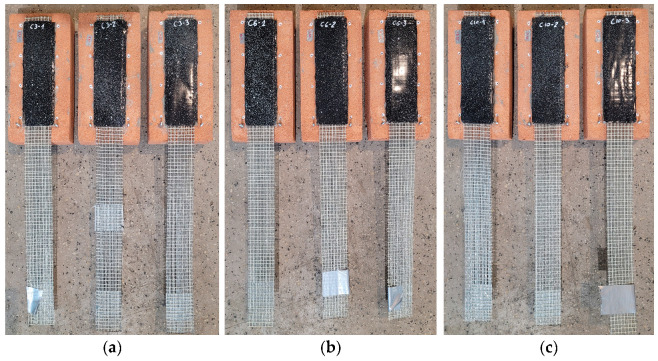

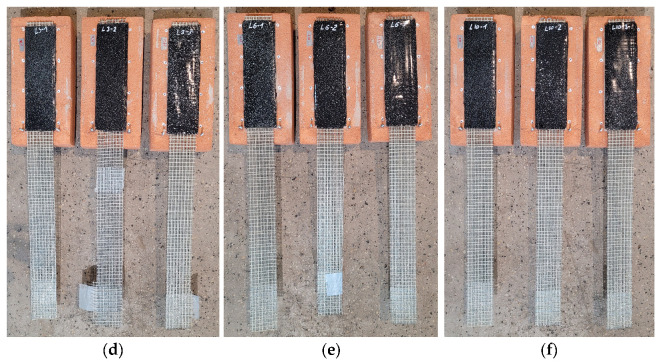
Specimens with mineral interlayer after SRPU application: (**a**) cement-based interlayer mortar of 3 mm thickness (C3); (**b**) cement-based interlayer mortar of 6 mm thickness (C6); (**c**) cement-based interlayer mortar of 10 mm thickness (C10); (**d**) lime-based interlayer mortar of 3 mm thickness (L3); (**e**) lime-based interlayer mortar of 6 mm thickness (L6); (**f**) lime-based interlayer mortar of 10 mm thickness (L10).

To perform single-lap shear tests, the specimens of the geometry given in Figure 3g were adopted. The preparation of the specimens was divided into two main stages: (i) application of the mortar interlayer on the substrate and (ii) application of the SRPU on the layer of the mortar. In the first step, bricks were wetted to avoid excessive absorption of water from the mortar (Figure 3a). Then, a layer of mortar of dimensions 200 mm in length and 60 mm in width was formed and cured (Figure 3b,c). After the curing period of 28 days, the mortar surfaces were cleaned with compressed air to remove any dust, followed by the application of a one-component polyurethane primer to enhance adhesion between the SRPU and the substrate (Figure 3d). Subsequently, a thin layer of polyurethane was applied over the mortar, and steel textile reinforcement was embedded using a steel scraper to ensure an adequate bond between the polyurethane and steel cords, as well as between the polyurethane and the mortar. Finally, a second layer of polyurethane was applied over the textile (Figure 3e,f). As the SRPU (for specimens L and C) was applied to the hardened mortar strips, the mortar became an interlayer between the brick and the SRPU. For reference specimens, the strip of SRPU was applied directly to the brick bed (R specimens), after the polyurethane primer was previously applied to the brick surface. The specimens, ready for SLSTs, are presented in Figure 4a–c and Figure 4d–f for cement- and lime-based interlayers, respectively.

### 2.3. Test Setup and Test Procedure

**Figure 5:**
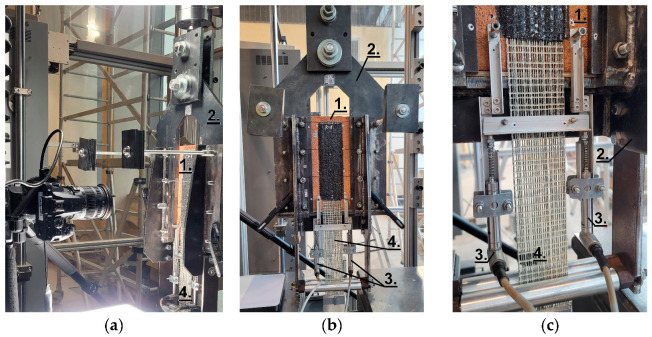
Test setup: (**a**) side view; (**b**) front view; (**c**) detail—LVDTs. 1. Tested specimen; 2. Supporting L-shaped steel frame; 3. LVDTs; 4. Loaded end of steel textile.

Shear bond tests were carried out seven days after SRPU application on three specimens of each type, as described in Table 1. The tests were performed using a single-lap push–pull configuration, described in detail in [31]. The specimens were positioned within a rigid frame to ensure they remained fixed during testing. The textile was clamped using wedges from the testing machine, which applied the pulling force. The tests were conducted under displacement control at a rate of 0.5 mm/min, continuing until detachment of the composite occurred. A pair of linear variable differential transformers (LVDTs) was used to measure the relative displacement (slip, s) at the loaded end of the bonded area. The applied load was measured using a 20 kN external load cell. A detailed view of the test setup and the arrangement of LVDTs is shown in Figure 5. Details of the test setup used here are presented in [26].

## 3. Results

The main results of the tests are summarized in Table 2, including the observed failure mode, peak force (F_max_), peak axial tensile stress in the steel textile (f_b_), average peak axial stress (f_b,av_), and corresponding coefficient of variation (CV). Additionally, for the L and C specimens, the strength ratio (SR) was determined as the ratio of the average peak axial stress (f_b,av_) of each specimen group to the reference value (f_b,av_) from the R specimens. The relationships between slip and axial force for the reference, C, and L specimens are presented in Section 3.1, Section 3.2, and Section 3.3, respectively. The slip (s) is the average value recorded by the pair of LVDTs.

### 3.1. Reference Specimens

**Figure 6:**
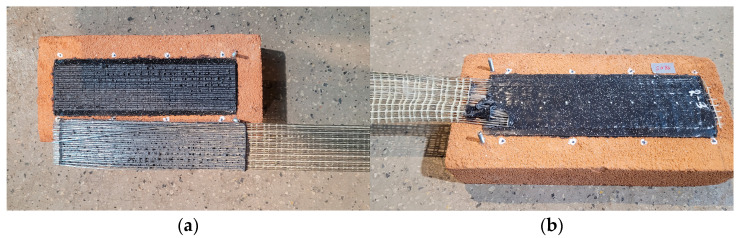
Failure modes observed in the tests of reference specimens: (**a**) failure mode FM5 (specimen R-2); (**b**) failure mode FM6 (specimen R-3).

**Figure 7:**
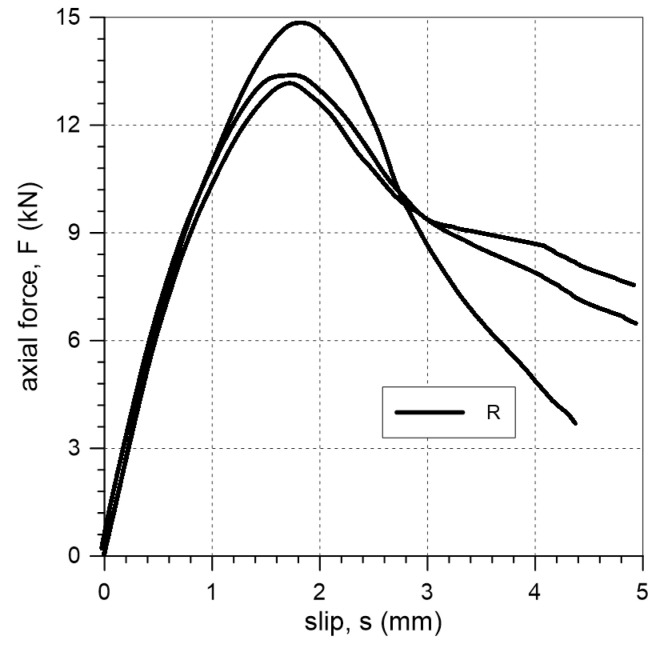
Slip–axial force curves of single-lap shear test for reference specimens.

Two failure modes were observed during tests on reference specimens: FM5 and FM6. Specimens R-1 and R-3 failed due to detachment at the interface between the matrix and the textile, while specimen R-2 failed due to sliding of the steel textile within the PS layer (Figure 6). The relationships between axial force and slip are presented in Figure 7. The maximum forces obtained in tests on R specimens ranged from 13.2 kN to 14.9 kN (Table 2), with corresponding slip values ranging from 1.7 mm to 1.8 mm (Figure 7). The reference specimens failed at a mean stress of 1069 N/mm^2^ (Table 2), which corresponds to 33% of the tensile strength of the steel textile.

### 3.2. Cement-Based Interlayer

**Figure 8:**
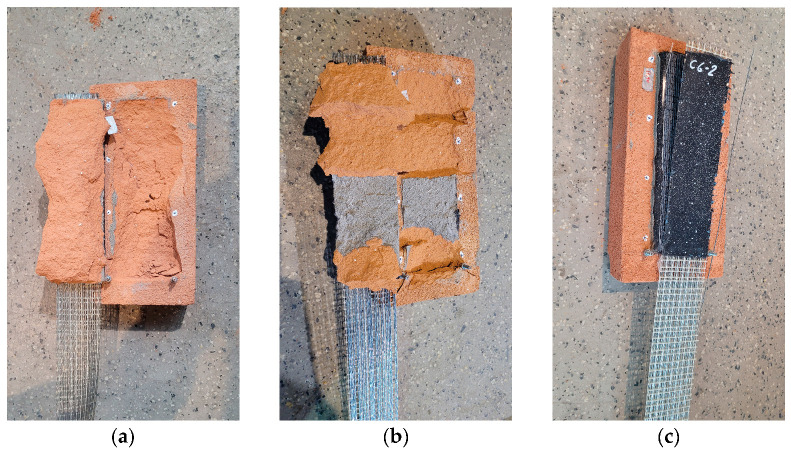
Failure modes observed in the tests of specimens with a cement-based interlayer: (**a**) failure mode FM1 (specimen C10-2); (**b**) failure mode FM1/FM2* (specimen C3-2); (**c**) failure mode FM5 (specimen C6-2).

**Figure 9:**
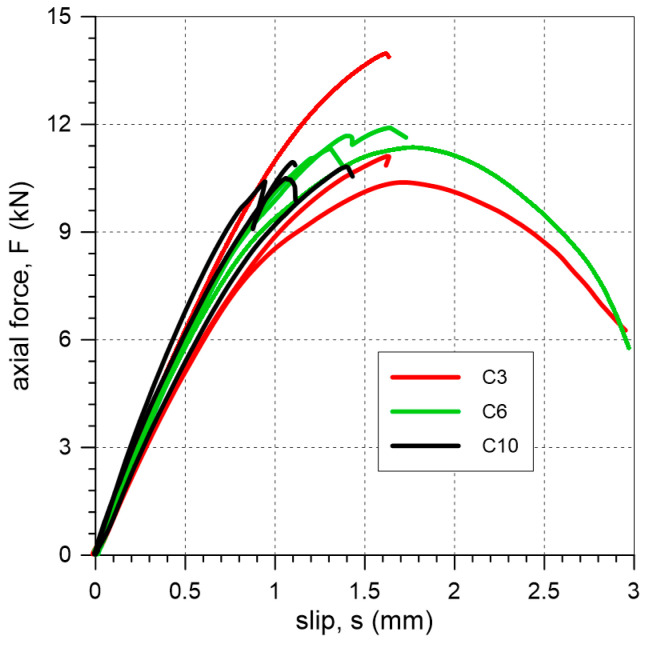
Slip–axial force curves of single-lap shear tests for specimens C.

The C specimens failed due to cohesive debonding within the substrate (clay brick) (FM1 mode) or due to detachment at the interface between the textile and the matrix (FM5 mode) (Figure 8). Axial force–slip curves from the tests are presented in Figure 9. The average values of maximum force/average slip obtained in tests on specimens with a cement interlayer were 11.8 kN/1.7 mm, 11.5 kN/1.6 mm, and 10.8 kN/1.2 mm for mineral interlayer thicknesses of 3 mm, 6 mm, and 10 mm, respectively.

### 3.3. Lime-Based Interlayer

**Figure 10:**
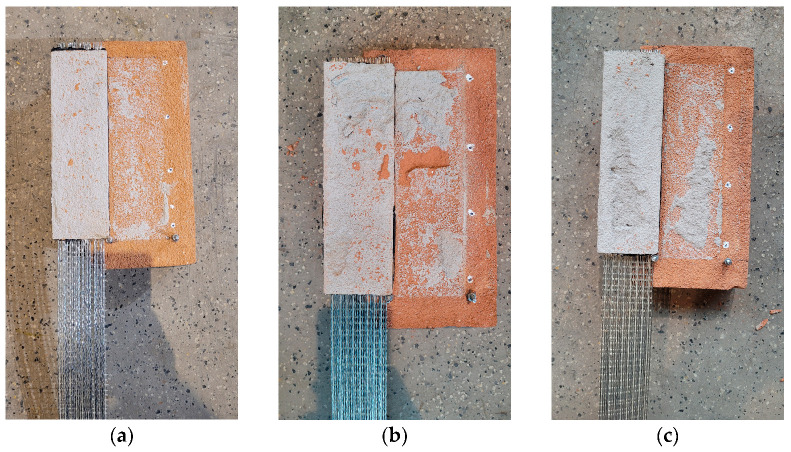
Failure modes observed in the tests of specimens with a lime-based interlayer: (**a**) failure mode FM3 (specimen L3-2); (**b**) failure mode FM3/FM2* (specimen L6-3); (**c**) failure mode FM3/FM1* (specimen L10-2).

**Figure 11:**
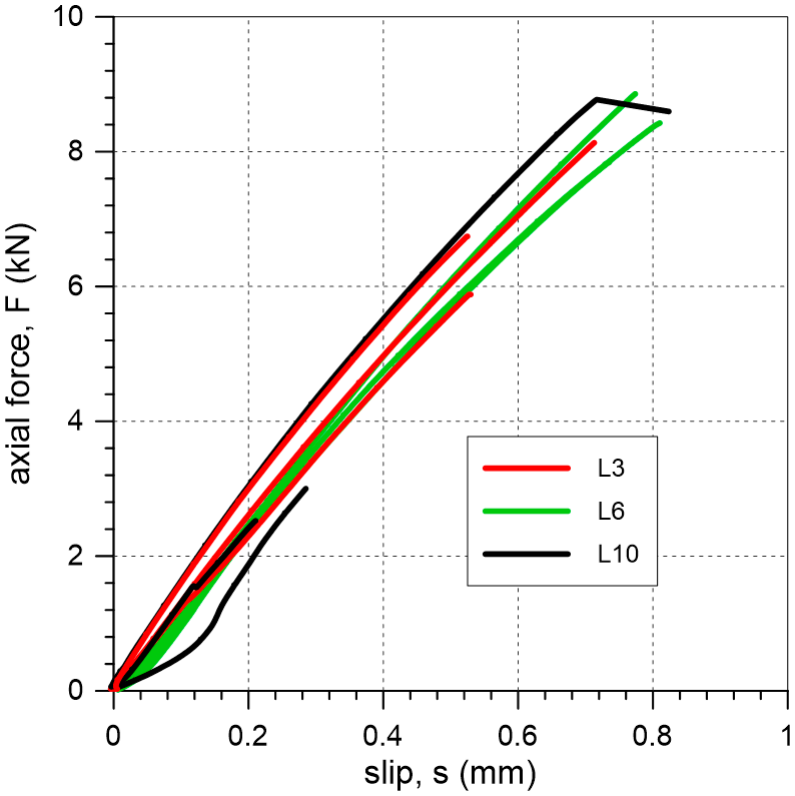
Slip–axial force curves of single-lap shear tests for SRPU bonded to brick substrate through lime-based mortar interlayer.

All specimens with lime mortar failed due to detachment of the mineral interlayer from the substrate: FM3 mode (Figure 10). In two cases, failure modes FM2 and FM1 were observed in a limited area: specimens L6-3 and L10-2. The axial force–slip curves are presented in Figure 11. For specimens L with a mineral interlayer thickness of 3 mm, the average maximum force and slip were 6.9 kN and 0.59 mm, respectively. For a 6 mm interlayer thickness, the corresponding values were 8.4 kN and 0.74 mm. Finally, for a 10 mm mineral interlayer, the average maximum force and slip were 4.8 kN and 0.41 mm, respectively.

## 4. Discussion

When strengthening of buildings in seismic areas is considered, it is crucial to select a method that ensures the structure has the ability to accommodate deformation without catastrophic failure in exceptional situations. Such characteristics are demonstrated by the analyzed FRPU system with a highly deformable polyurethane PS matrix. In the tests, when the composite was applied directly to the brick—specimens R—the axial force–slip curve did not exhibit brittle characteristics (Figure 7). In these tests, the failure occurred in the polyurethane matrix; failure modes FM5 and FM6 were observed (Figure 6).

In the case of specimens with a cement interlayer (C), the applied load was effectively transferred from the steel cords to the clay brick. In most cases, the observed failure occurred within the brick (failure mode FM1) (Figure 8). This failure mode was also commonly observed in FRP or SRP composites [1]. The observed FM1 failure mode suggests that a strong mineral plaster could be a contributing factor to the development of significant damage in the masonry. The curves obtained from the tests of specimens C3-1 and C6-2 (Figure 9), which failed due to detachment between the textile and polyurethane, exhibit post-peak behavior. The curves of the other specimens confirm the brittle nature of the failure: these specimens failed suddenly due to cohesive debonding within the substrate.

In the case of specimens with a lime interlayer (L), the applied load was transmitted from the reinforcement to the brick, ultimately resulting in detachment at interface between the mortar and the brick (failure mode FM3) (Figure 10). This failure mode is typical of mineral matrices with low bond strength, where stress concentrations occur between two brittle materials. The observed FM3 failure mode suggests that the use of a weak lime mortar interlayer leads to a failure mechanism that is safer for the preservation of heritage substrate than those observed in tests on the C specimens. The slip–axial force curves for all the specimens appear to be linear over the entire range of loading; no post-peak behavior was observed in those cases (Figure 11).

**Figure 12:**
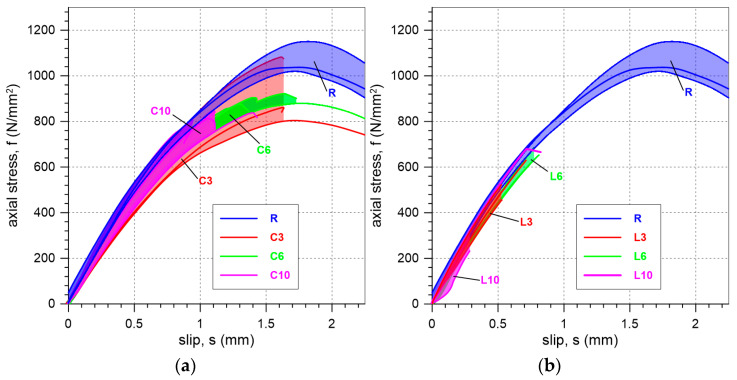
Comparison of slip–axial stress curves obtained in the tests of (**a**) specimens with a cement-based interlayer, and (**b**) specimens with a lime-based interlayer.

**Figure 13:**
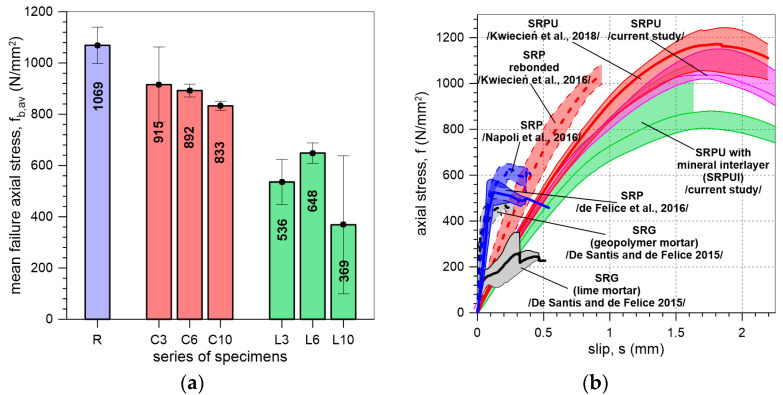
(**a**) Bar graph illustrating the mean and standard deviation (error bars) of failure axial stress obtained in the tests. (**b**) The results of previous and current single-lap shear tests on externally bonded steel-reinforced composite systems for masonry protection [11,12,14,26,27].

Comparing the results for specimens with the mineral interlayers used (C and L) to those for reference specimens (R), it can be observed that the presence of the interlayer led to a reduction in the maximum stress that the bond can carry (Figure 12 and Figure 13a). The strength ratios of 86%, 84%, and 78% for cement mineral interlayers with thicknesses of 3 mm, 6 mm, and 10 mm, respectively, indicate a slightly lower efficiency of the system in terms of load transfer compared to specimens without an interlayer (Table 2). Even lower efficiency is observed for the lime interlayer, where the strength ratios for specimens with 3 mm, 6 mm, and 10 mm thick mineral interlayers were found to be 50%, 61%, and 35%, respectively (Table 2). The higher bond strength of the reference specimens compared to the specimens with a mineral interlayer is due to the uniform distribution of stress between the SRPU and the substrate [26]. The use of an additional mineral mortar layer generates stress concentrations at the mortar–brick interface, which is why failure is most commonly observed at the interlayer–brick interface.

The influence of mineral interlayer thickness on the maximum axial stress obtained in tests is shown in Figure 13a. For specimens C, the thicker the interlayer, the lower the maximum load recorded. For specimens L, the trend is not clear, which may be due to the limited number of specimens and the high coefficient of variation (CV), especially for the L10 specimens.

The presented axial stress–slip relationships may be useful in the design and analysis of the potential application of the tested solutions on existing structures (Figure 12). In such analyses, it can be assumed that the stiffness (the slope of the axial stress–slip curve) of the bond under shear is approximately 0.8 kN/mm³ for all the tested specimens (R, L, and C). This assumption can be made because the flexible polyurethane matrix primarily governs the behavior of the system in all the considered cases—both with and without a mineral interlayer—regardless of the thickness of the mineral layer and its strength. This is due to the fact that polyurethane (PS) has a modulus of elasticity of 14.8 MPa, in contrast to the much higher moduli of the mortars used, which are 16 GPa for lime mortar and 10 GPa for cement mortar. This significant difference in stiffness explains why the overall shear stiffness of the analyzed system is mainly governed by the polyurethane.

In Figure 13b, SLST results of the present and previous studies on composites reinforced with steel textile are compared. The previous studies concerned specimens of different systems with different matrices bonded to clay brick or brickwork, such as SRP [11,12] composites with epoxy matrices; steel-reinforced grout (SRG) systems with NHL lime or geopolymer mortar [14]; systems with polyurethane of PS type; SRPU [26]; and rebonded SRP [27]. The curves for SRPU systems clearly demonstrate that the shear stiffness is very similar across all tested specimens, suggesting that the behavior of the specimens is primarily governed by the polyurethane used. A comparison with current research confirms that SRPU systems are more effective in utilizing the steel reinforcement. However, it is important to note that the results presented are based on a bond length of 200 mm. The increase in the bond length results in an increase in the shear bond strength, which leads to a higher strength ratio of the steel textile (the ratio between maximum axial stress in steel cords and the tensile strength of the textile). SRPU systems demonstrate more than double the peak axial stress in the SLST compared to the other systems. This is attributed to the uniform distribution of stress [26] and reduction in stress concentrations within the flexible matrix, which enhances the ability of the SRPU system to safely withstand higher loads. Additionally, when comparing results for SRG and SRP with SRPU (Figure 13b), it can be noticed that composites with a polyurethane matrix exhibit less stiff and more ductile behavior, which is advantageous in seismic regions. This is particularly beneficial when combined with the excellent energy dissipation capabilities of the polyurethane matrix, which has viscoelastic properties.

In this paper, SRPUI systems with lime or cement interlayer mortars were tested. The mortars used here had compressive strengths of 7.2 N/mm^2^ and 32.6 N/mm^2^, respectively. A comparison of the SRPUI system with a lime interlayer to SRG systems with a lime matrix (compressive strength of 20.6 N/mm^2^) shows that the loads carried by specimens L were nearly twice as high as those for SRG specimens. This is despite the fact that the compressive strength of the matrix used in the steel-reinforced grout system was nearly three times greater than that of the mortar used in SRPUI (Figure 13b). When a similar comparison is made between the SRPUI system with a cement interlayer and SRG with a geopolymer mortar of compressive strength 56.3 N/mm^2^, it can be observed that specimens C exhibit nearly twice the ultimate stress compared to SRG systems using geopolymer mortar (Figure 13b). This is even though the compressive strength of the matrix in the steel-reinforced grout system is nearly twice as great as that of the mortar used in SRPUI (Figure 13b). There is more uniform stress distribution and reduction in stress concentrations within the polyurethane matrix in SRPUI systems.

Additionally, it is worth noting that in steel-reinforced grout systems, mineral mortars of high strength are used. Attempts to remove such materials from the face of a structural element may cause damage to the surface of the structure on which the composite was applied. However, in the case of systems with polyurethane matrices (SRPU and SRPUI), the composite can be easily removed without damaging the historic substrate [30,35].

Based on the above results, further investigations are needed to fully understand the behavior of FRPU strengthening systems with mineral interlayers. Future research should focus on exploring the effects of different fiber grid types, mortar compositions, bond lengths, and polyurethane types on shear bond strength. Additionally, studies should consider a broader range of heritage substrates or their substitutes to better assess the versatility and applicability of these strengthening systems in real-world restoration scenarios.

The tested system reinforced with steel textile demonstrated notable efficiency in monotonic testing, surpassing previously reported results in the literature. This enhanced performance is primarily attributed to the effective distribution of shear stresses within the flexible polyurethane matrix. It is important to highlight that similar positive outcomes are anticipated when applying this system to masonry structures in seismic zones, where higher loads and significant deformations are common during earthquakes. This prediction is supported by earlier seismic studies on FRPU systems with glass fibers—significantly weaker than steel fibers—which successfully withstood repeated dynamic loads and large deformations [22,23,24].

## 5. Conclusions

This paper presents the outcomes of single-lap shear tests conducted on an SRPUI composite system with a mineral interlayer for the protection of masonry structures. The key findings drawn from this study are as follows:

- The strength obtained in the test on the bond between the SRPUI composite and the brick substrate was strongly influenced by the mechanical properties of the material used as the interlayer. An increase in shear bond strength was observed for the cement interlayer compared to the lime interlayer. The ratios of maximum axial stress for specimens with a cement interlayer to those with a lime interlayer were 171%, 138%, and 225% for mortar interlayer thicknesses of 3 mm, 6 mm, and 10 mm, respectively. This improvement is attributed to the higher bond, compressive, and tensile strength of the cement mortar. However, the presence of the interlayer led to a reduction in shear bond strength of up to 22% for the cement interlayer with a thickness of 10 mm, and a 65% reduction for the lime interlayer with a thickness of 10 mm, when compared to the reference specimens without an interlayer.
- Regardless of the applied mortar, the shear bond strength of the tested SRPUI systems is influenced by the thickness of the mortar interlayer. For cement mortar, an increase in interlayer thickness resulted in a reduction in shear bond strength. In the case of lime mortar, the lowest strength was recorded for the thickest layer (10 mm); however, the trend was not consistent. It may be attributed to the limited number of specimens and the high coefficient of variation.
- The mechanical properties of the applied material influence the observed failure modes. For specimens with a cement interlayer, cohesive debonding within the substrate was the dominant failure mode, while for specimens with a lime interlayer, failure occurred at the interface between the interlayer and the brick.
- The stiffness of the bond under shear in the tested SRPUI systems was unaffected by the presence of the mineral interlayer. Despite the varying mechanical properties of the materials used as the interlayer, similar stiffness values were observed across all the specimens. This can be attributed to the high deformability of the polyurethane matrix and the relatively rigid nature of the mineral interlayer.

## Figures and Tables

**Table 1:** Tested specimens.

Notation	No. of Specimens	Interlayer	Interlayer Thickness (mm)
R	3	-	-
C3	3	Cement-based	3
C6	3	Cement-based	6
C10	3	Cement-based	10
L3	3	Lime-based	3
L6	3	Lime-based	6
L10	3	Lime-based	10

**Table 2:** Test results.

Notation	Failure Mode	F_max_ (kN)	f_b_ (N/mm^2^)	f_b,av_ (N/mm^2^)
R-1	FM6	13.4	1037	1069
R-2	FM5	14.9	1150	CV 6.6%
R-3	FM6	13.2	1019	SR 100%
C3-1	FM5	10.4	804	915
C3-2	FM1/FM2 *	11.1	860	CV 16.1%
C3-3	FM1	14.0	1082	SR 86%
C6-1	FM1	11.9	922	892
C6-2	FM5	11.4	880	CV 2.8%
C6-3	FM1	11.3	876	SR 84%
C10-1	FM1	10.5	813	833
C10-2	FM1	10.8	838	CV 2.1%
C10-3	FM1	10.9	847	SR 78%
L3-1	FM3	5.88	455	536
L3-2	FM3	8.13	630	CV 16.4%
L3-3	FM3	6.74	522	SR 50%
L6-1	FM3	7.84	607	648
L6-2	FM3	8.86	686	CV 6.1%
L6-3	FM3/FM2 *	8.42	652	SR 61%
L10-1	FM3	8.77	679	369
L10-2	FM3/FM1 *	3.00	232	CV 73.0%
L10-3	FM3	2.52	195	SR 35%

CV—coefficient of variation; SR—strength ratio; *—observed on limited area.

## Data Availability

The original contributions presented in this study are included in the paper; further inquiries can be directed to the corresponding author.
